# Optimization of Ultrasonic Assisted Extraction of Antioxidants from Black Soybean (*Glycine max* var) Sprouts Using Response Surface Methodology

**DOI:** 10.3390/molecules18011101

**Published:** 2013-01-16

**Authors:** Jixiang Lai, Can Xin, Ya Zhao, Bing Feng, Congfen He, Yinmao Dong, Yun Fang, Shaomin Wei

**Affiliations:** 1 School of Chemical and Material Engineering, Jiangnan University, Wuxi 214122, Jiangsu, China; 2 School of Chemical Engineering, Hebei United University, Tangshan 063009, Hebei, China; 3 Shanghai Jahwa United Co. Ltd, Shanghai 200082, China; 4 School of Science, Beijing Technology and Business University, Beijing 100048, China

**Keywords:** black soybean sprouts, antioxidant, ultrasonic assistedextraction, aqueous extraction, response surface methodology

## Abstract

Response surface methodology (RSM) using a central composite design (CCD) was employed to optimize the conditions for extraction of antioxidants from black soybean (*Glycine max* var) sprouts. Three influencing factors: liquid-solid ratio, period of ultrasonic assisted extraction and extraction temperature were investigated in the ultrasonic aqueous extraction. Then Response Surface Methodology (RSM) was applied to optimize the extraction process focused on DPPH radical-scavenging capacity of the antioxidants with respect to the above influencing factors. The best combination of each significant factor was determined by RSM design and optimum pretreatment conditions for maximum radical-scavenging capacity were established to be liquid-solid ratio of 29.19:1, extraction time of 32.13 min, and extraction temperature of 30 °C. Under these conditions, 67.60% of DPPH radical-scavenging capacity was observed experimentally, similar to the theoretical prediction of 66.36%.

## 1. Introduction

Black soybean (*Glycine max* var) is the black seed of the soybean (*Glycine max* (L.) *merr*), also known as the black bean. It is mainly cultivated in the provinces of Shanxi and Hebei for both food and medicinal purposes. The *Supplement to Compendium of Materia Medica* states that black beans can be beneficial to sperm and bone marrow production, muscle strength, hair growth, and the immune system. Modern scientific research shows that black beans have hypolipidemic and antioxidant properties and can be used to beautify the skin [1]. According to the updated research results, the antioxidants in black soybeans were pigments [2], polypeptides [3] and anthocyanin compounds [4]. Studies have shown that more antioxidants can be extracted from the bud of germinated black beans. The proteins, polysaccharides and mineral elements in sprouts are released by enzyme activity, resulting in greater antioxidant properties [5,6,7]. Black soybean sprout extract is a green and healthy antioxidant, safe to use as additive in anti-aging and whitening cosmetic products [8]. In order to increase the safety of products and avoid skin irritation, deionized water was used as extraction solvent. Secondly, polypeptides are the the major antioxidants in black soybean sprout [3], and are readily soluble in water.

Many different techniques have been employed for the extraction of antioxidants from plants. The conventional extraction methods, like reflux extraction and maceration extraction have many drawbacks, including the need for long extraction times and the need for relatively large quantities of solvent [9]. In addition, most active ingredients of plants are found in their cells, making it difficult for mechanical crushers to break cells for extraction, and chemical crushing methods may damage the active molecules and inactivate the extract. Compared with conventional and other modern extraction techniques, ultrasonic assisted extraction (UAE) is proposed as an alternative procedure for sample pretreatment and as a greener methodology that allows for a high reproducibility in shorter time, simplified manipulation, significant reduction in organic solvent consumption and temperature, and lower energy input [10,11]. Especially, UAE has great advantages in the extraction of polar compounds using water as solvent [12].

Response surface methodology (RSM) is a kind of optimization method which serves as an accurate, effective, and simple tool for optimizing the experimental process [13,14], and is widely used in agriculture, biology, food, chemistry and other fields [15,16,17]. There is little available literature about optimization of the antioxidants extraction process from black soybean sprouts using RSM. This present study focuses on UAE of antioxidants in black soybean sprouts; three influencing factors in the aqueous extraction of antioxidants such as liquid-solid ratio, period of UAE and extraction temperature were investigated. Taking DPPH radical scavenging capacity of antioxidants as the index, the extraction processing was optimized using RSM on the basis of the single factor method.

## 2. Results and Discussions

### 2.1. Results of Single-Factor Experiments

**Figure 1:**
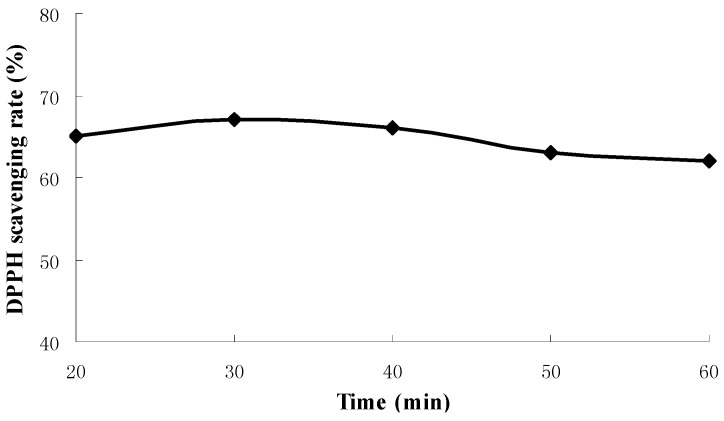
The influence of extraction time on antioxidant activities of extraction.

**Figure 2:**
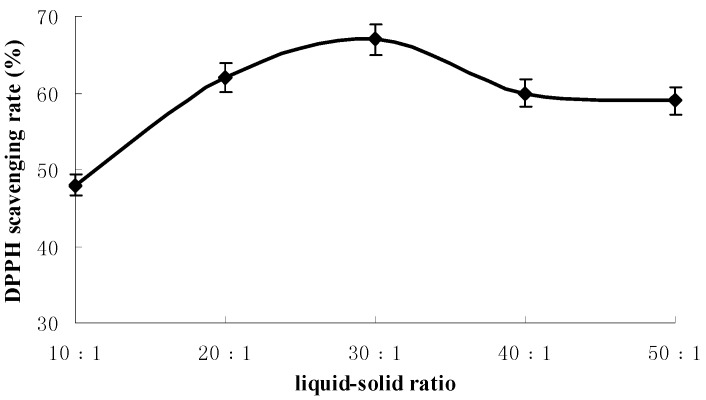
The influence of liquid-solid ratio on antioxidant activities of extraction.

**Figure 3:**
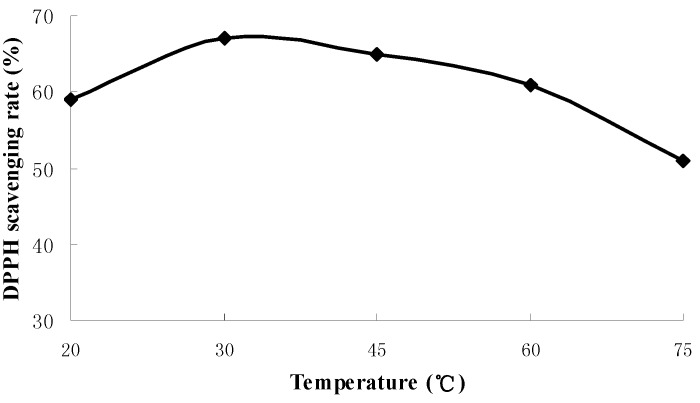
The influence of temperature on antioxidant activities of extraction.

The influences of extraction time, liquid-solid ratio and extraction temperature on antioxidant activities of extraction were shown in Figure 1, Figure 2 and Figure 3 respectively.

It can be seen from Figure 1 that DPPH scavenging rates reached more than 65% after 30 min and about 60% after 60 min, there was no significant difference of scavenging rates for extraction times of 20 to 60 min. Extracts of 30 min had the highest scavenging rate at various times.

Figure 2 shows that the liquid-solid ratio which gave the highest DPPH radical scavenging rate was at 30:1 mL/g. The scavenging rate increase with liquid-solid ratio from 10:1 to 30:1 and reduces from 30:1 to 50:1. Therefore, the most appropriate liquid-solid ratio is 30:1.

Figure 3 shows that DPPH radical scavenging rate increased with temperature from 20 to 30 °C and was reduced from 30 to 75 °C. The highest scavenging rate was obtained at the temperature of 30 °C, possibly due to the components with anti-oxidizing capacity have been mostly extracted at 30 °C and some antioxidants were destroyed at higher temperatures. Therefore, 30 °C is a suitable extraction temperature. From all these results, the effective experimental ranges selected for the RSM study were an extraction time of 20 to 40 min, liquid-solid ratio from 20:1 to 40:1 and extraction temperature from 30 to 60 °C.

### 2.2. Results of Response Surface Methodology Experiments

The effect of three independent variables, namely, extraction temperature (A), extraction time (B), liquid-solid ratio (C) on DPPH radical scavenging activity (Y) was investigated using a three-factor CCD-RSM experimental and the results are shown in Table 1.

**Table 1:** Response surface analysis program and results for black beans sprout extract.

Run	Factor1	Factor2	Factor3	Clearance rate
	A: Temperature (°C)	B: Time (min)	C: liquid-solid ratio	Y: (%)
1	30	40	30:1	66.52
2	45	30	30:1	67.56
3	60	20	30:1	62.98
4	60	30	20:1	62.43
5	30	20	30:1	65.28
6	45	20	40:1	61.37
7	45	20	20:1	60.38
8	60	30	40:1	60.88
9	45	30	30:1	65.57
10	30	30	40:1	60.10
11	45	30	30:1	65.55
12	30	30	20:1	62.75
13	45	40	20:1	60.17
14	60	40	30:1	61.25
15	45	40	40:1	60.77
16	45	30	30:1	65.25
17	45	30	30:1	65.55

After data fitting to the experimental findings, one gets the following regression equation: Y = 13.3375 + 0.23845A + 1.06255B + 2.22142C − 0.04950AB + 0.01833BC − 0.09750AC − 0.02269A^2^ − 0.01378B^2^ − 0.03845C^2^. Analysis of variance for response surface quadratic model was shown in Table 2.

As shown in Table 2, the Model F-value of 4.21 implies the model is significant. There is only a 3.57% chance that a “Model F-Value” this large could occur due to noise. Values of “Prob > F” less than 0.0500 indicate model terms are significant. In this case C^2^ are significant model terms. Values greater than 0.1000 indicate the model terms are not significant. There are many insignificant model terms (not counting those required to support hierarchy), so model reduction may improve the model.

**Figure 4:**
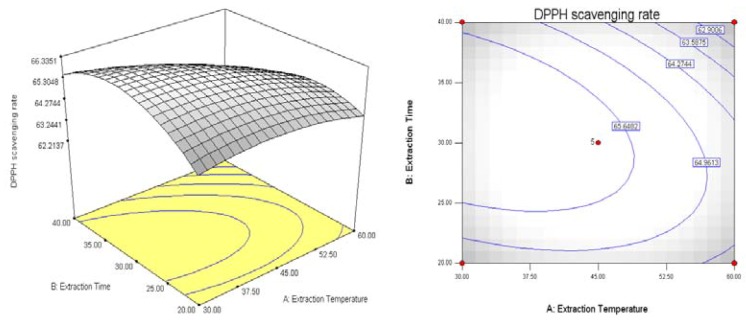
Response surfaces and contour plots showing the effects of extraction temperature and extraction time on antioxidant activity Y = (A, B).

**Figure 5:**
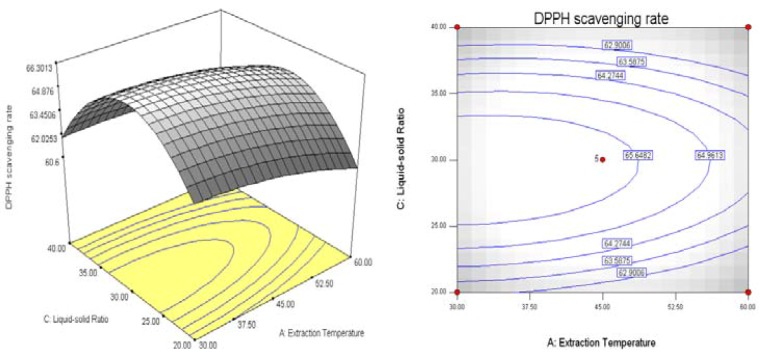
Response surfaces and contour plots showing the effects of extraction temperature and liquid-solid ratio on antioxidant activity Y = (A, C).

**Figure 6:**
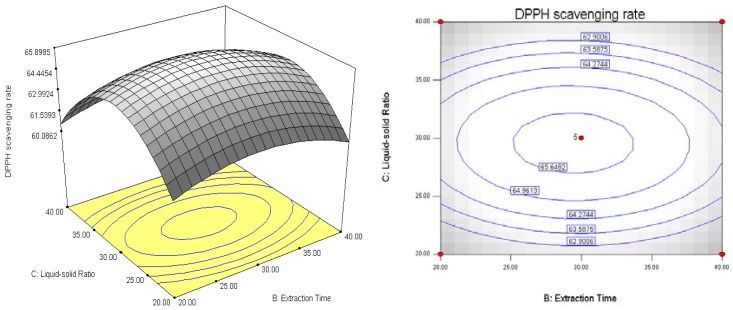
Response surfaces and contour plots showing the effects of extraction time and liquid-solid ratio on antioxidant activity Y = (B, C).

The analyses of the data were done using the Design-Expert 7.1 software. Response surfaces and contour plots were shown in Figure 4, Figure 5 and Figure 6. The interaction between various factors can be seen directly from the response surfaces and contour plots. The contour plot in Figure 4 is flat, indicating the interaction between extraction temperature and extraction time affects more the antioxidant activity of the extract. Similarly, the contour plot in Figure 5 indicates the effects of extraction temperature and liquid-solid ratio on antioxidant activity is bigger. The contour plot in Figure 6 is round, which implies the the interaction between extraction time and liquid-solid ratio has a minimum effect on the antioxidant activity of the extract. 

**Table 2:** Analysis of variance for response surface quadratic model.

Source	Sum of Squares	DF	Mean Square	F Value	Prob > F	Significance
Model	85.45	9	9.49	4.21	0.0357	significant
A	6.32	1	6.32	2.80	0.1382	
B	0.21	1	0.21	0.094	0.7685	
C	0.85	1	0.85	0.38	0.5584	
A^2^	1.10	1	1.10	0.49	0.5081	
B^2^	8.00	1	8.00	3.54	0.1018	
C^2^	62.26	1	62.26	27.59	0.0012	
AB	2.21	1	2.21	0.98	0.3558	
AC	0.30	1	0.30	0.13	0.7251	
BC	0.038	1	0.038	0.017	0.9004	
Residual	15.80	7	2.26			
Lack of Fit	12.26	3	4.09	4.63	0.0864	Not significant
Pure Error	3.53	4	0.88			
Cor Total	101.25	16				

The response surfaces and contour plots of Figure 4, Figure 5 and Figure 6 show that the extraction time did not have a significant effect on antioxidant activity. This result implies that 30 min of extraction time is sufficient for the antioxidants from black soybean sprout to dissolve in deionized water. Another important observation from this study was that the antioxidant activity increases as the liquid-solid ratio decreases, while the extraction temperature did have a positive effect on antioxidant activity, when extraction time was held constant at 30 min. The increase of antioxidant activity with decrease of liquid-solid ratio can be explained by mass transfer. The higher the liquid-solid ratio, the greater the driving force within the solid, which results the increase of diffusion rate. The main effect of the liquid-solid ratio was to modify the solubility and equilibrium constants and thus increase the antioxidants to a maximum at a lower liquid-solid ratio [18]. Antioxidant activity was reduced with increased extraction temperature. This may be due to the fact the antioxidants were destroyed at higher temperatures.

To get the optimized value of the extraction conditions, the three independent variables, namely, extraction temperature (A), extraction time (B), liquid-solid ratio (C) were transformed as follows: X_1_ = (A−45)/15, X_2_ = (B−40)/10, X_3_ = (C−30)/10. The regression equation transfer into: Y = − 405.5275 − 100.62975X_1_ − 17.1745X_2_ −103.725X_3_ − 5.10525X_1_^2^ −1.378X_2_^2^− 3.845X_3_^2^ − 7.425X_1_X_2_ + 1.833X_2_X_3_ − 14.625X_1_X_3_.

The optimal conditions analyzed using Design Expert 7.1 were as follows: 30 °C of extraction temperature, 32.13 min of extraction time, and 29.19:1 of liquid-solid ratio. Under optimal conditions, the theoretical value of DPPH radical scavenging rate of black soybean sprout extract was 66.36%, and the measured actual scavenging rate was 67.60%, very close to the theoretical prediction. The results show that response surface methodology is a useful mathematical tool in finding the optimum extraction conditions for antioxidants from black soybean sprouts.

## 3. Experimental

### 3.1. Materials and Reagents

The following reagents and materials were obtained for this study: soybeans (*Glycine max* var) (Shijiazhuang, China); 1,1-diphenyl-2-picrylhydrazyl radical (DPPH•), AR (Biodee Biotechnology Co. Ltd, Beijing, China); Vitamin C, AR (Beijing Chemical Reagent Company, Beijing, China).

### 3.2. Sample Preparation

Black soybean (*Glycine max* var, Hebei Province) was used in this investigation. Five grams of soybean seeds were soaked in distilled water (200 mL) for 16 h and then placed in a germinator (model QFR-A801, Zhejiang Xingda Co., Ltd., Jinhua, China). Germination occurred at 25 °C and 99% relative humidity in darkness with water sprayed every 60 min [19,20].

### 3.3. Antioxidants Extraction

Black bean sprouts were extracted according to the method described in references [21,22,23,24,25]. Five grams of dry black soybeans were germinated to 0.5 cm and then distilled water was added in accordance with a certain liquid-solid ratio(10:1–50:1, crushed using a flash extractor (JHBE-50T, Taikang Biotechnology Inc., Xi’an, China) for 3 min at the 40 V voltage, extracted by ultrasonification at 40 KHz for 20–60 min at different temperatures (20–75 °C). The extract was produced by filtration of the crude sample through a two-layer cheesecloth filter. The filtrate was centrifuged (Sigma 4K-15, Goettingen, Germany) at 10,000 rpm for 15 min at 20 °C, after which the supernatant was collected to freeze-dried (VIRTIS-K, Virtis Corporation, New York, NY, USA) to get the black bean sprout antioxidant lyophilized powder.

### 3.4. Determination of the Antioxidant Capacity

Antioxidant activity can be measured by different methods, such as superoxide dismutase-like activity (SOD-like activity), peroxyl radical-trapping capacity (PRTC), Trolox equivalent antioxidant capacity (TEAC) and DPPH radical scavenging capacity (DPPH•). DPPH• is a stable organic free radical which can be used to give a global measure of antioxidant activity by spectrophotometry; therefore, antioxidant activity of extracts were measured using DPPH• scavenging method [26]. Ethanol solution of DPPH· (1 mM, 3 mL) were mixed with sprout extract (3 mL), centrifuged for 10 s, and then incubated for 30 min at 37 °C. Vitamin C and distilled water were used as positive control and blank control, respectively. After incubation, samples were centrifuged for 30 s at 13,000 rpm (room temperature). The absorbance of each sample at 517 nm was measured. Antioxidant activity is given as the percentage (%) of DPPH scavenging rate, calculated as: (absorbance of blank control—absorbance of extract)/absorbance of blank control × 100%. The results were recorded in the mean of three independent experiments.

### 3.5. Single-Factor Experiments

A screening study was conducted to determine appropriate ranges of independent variables, *i.e.*, extraction time, liquid-solid ratio and extraction temperature to be used in design of experiments. It involved extraction times of 20, 30, 40, 50, 60 min at fixed liquid-solid ratio of 30:1 (30 mL of deionized water to 1 g powder) and extraction temperature of 30 °C. Then, liquid-solid ratio was evaluated at different ratios from 10:1 to 50:1 at a fixed extraction time of 30 min and extraction temperature of 30 °C. Extraction temperature was evaluated from 20 to 75 °C at a fixed extraction time of 30 min and liquid-solid ratio of 30:1.

### 3.6. Response Surface Methodology Experiments

Response surface methodology was employed to establish the optimum conditions for extracting antioxidants from black soybean sprout. The effect of three independent variables, namely, extraction time (20–40 min), liquid-solid ratio (20:1–40:1), extraction temperature (30–60 °C) on DPPH radical scavenging activity was investigated using a three-factor CCD-RSM experimental runs to determine the optimal parameters of the extraction process [27,28,29,30]. Experimental factors and levels are shown in Table 3.

**Table 3:** Factors and levels of response surface analysis.

Levels	Independent variables		
	*A* Temperature/°C	*B* Time/min	*C* Liquid-solid ratio
−1	30	20	20:1
0	45	30	30:1
1	60	40	40:1

### 3.7. Statistical Methods

The analyses of the data were done using the Design-Expert 7.1. (Stat-Ease, Inc. Minneapolis, MN, USA).

## 4. Conclusions

In this study, RSM was used to investigate the main and interaction effects of important independent variables for extraction of antioxidants from black soybean sprouts on the basis of single-factor experiments. The optimum conditions for antioxidant extraction from black soybean sprouts were established. Research shows that the optimized experimental process increases the antioxidant activity of antioxidants from black soybean sprouts. The manufacturing process optimized using RSM needs lower energy, shorter time and offers simplified manipulation. The extract was green, healthly, safe and thus shows potential as an additive in cosmetic products. In addition, the successful extraction of antioxidants from black soybean sprout provides a basis for the development and utilization of black soybean resources.
